# Distribution and predictors of emergency department charges: the case of a tertiary hospital in Lebanon

**DOI:** 10.1186/s12913-016-1337-2

**Published:** 2016-03-18

**Authors:** Shadi Saleh, Yara Mourad, Hani Dimassi, Eveline Hitti

**Affiliations:** Department of Health Management and Policy, Faculty of Health Sciences, American University of Beirut, Beirut, Lebanon; School of Pharmacy, Lebanese American University, Beirut, Lebanon; Department of Emergency Medicine, Faculty of Medicine, American University of Beirut Medical Center, Beirut, Lebanon

**Keywords:** Emergency department, Charges, Cost, Lebanon, Cost categories

## Abstract

**Background:**

As health care costs continue to increase worldwide, health care systems, and more specifically hospitals are facing continuous pressure to operate more efficiently. One service within the hospital sector whose cost structure has been modestly investigated is the Emergency Department (ED). The study aims to report on the distribution of ED resource use, as expressed in charges, and to determine predictors of/contributors to total ED charges at a major tertiary hospital in Lebanon.

**Methods:**

The study used data extracted from the ED discharge database for visits between July 31, 2012 and July 31, 2014. Patient visit bills were reported under six major categories: solutions, pharmacy, laboratory, physicians, facility, and radiology. Characteristics of ED visits were summarized according to patient gender, age, acuity score, and disposition. Univariate and multivariate analyses were conducted with total charges as the dependent variable.

**Results:**

Findings revealed that the professional fee (40.9 %) followed by facility fee (26.1 %) accounted for the majority of the ED charges. While greater than 80 % of visit charges went to physician and facility fee for low acuity cases, these contributed to only 52 and 54 % of the high acuity presentations where ancillary services and solutions’ contribution to the total charges increased. The total charges for males were $14 higher than females; age was a predictor of higher charges with total charges of patients greater than 60 years of age being around $113 higher than ages 0–18 after controlling for all other variables.

**Conclusion:**

Understanding the components and determinants of ED charges is essential to developing cost-containment interventions. Institutional modeling of charging patterns can be used to offer price estimates to ED patients who request this information and ultimately help create market competition to drive down costs.

## Background

As health care costs continue to increase worldwide, health care systems are facing continuous pressure to operate more efficiently [1]. Since hospitals constitute the largest cost item within the system in most countries, particular attention has been focused on their operations vis-à-vis financial consistency and efficiency [2, 3]. One service within the hospital sector whose cost structure has been modestly investigated is the Emergency Department (ED). This is despite the common belief, supported by evidence, which identified EDs as a potential contributor to cost inefficiency within the health care system [4, 5]. That belief is based on the nature of the service within EDs; providing emergency health care and stabilizing the acute health conditions of patients on a 24-hour basis availability [6, 7]. Such a care structure is frequently associated, for many reasons including staffing, infrastructure and operational model, with elevated prices for minor procedures as compared to that charged in other ambulatory care settings [8–12]. That is why the increase in ED services’ costs associated with increased use, especially among individuals who are publicly insured and low-income population groups, is of concern due to the potential financial burden on the system [13–15].

Cost of emergency care has increased tremendously over the past decade [16, 17] with an estimated 240 % increase has been reported [16]. More strikingly is the variation in ED charges across institutions [11, 18]. A study has shown that out-of-pocket fees for the emergency treatment of sprains and strains, for example, ranged from $4 to $24,110 [11]. With the global trend of increasing ED utilization and associated costs, various cost containment solutions are being proposed in the literature including price transparency and financial counseling in the ED [1, 16, 19]. Such initiatives make understanding the structure of ED costs of paramount importance. However, few studies have investigated such an important topic, especially examining the resource use distribution in ED visits and potential contributors to it [2, 3, 19–21].

The paper is based on the argument that as hospital administrations are faced with growing pressure to achieve higher levels of cost efficiency, a clear understanding of the components of and contributors to a key service resource use, ED, becomes essential. This is particularly relevant in a region where the existence and use of evidence to guide decision-making is limited. The purpose of this study was to report on the distribution of ED resource use, as expressed in charges, by expense category and to determine predictors of/contributors to total ED charges at a major tertiary hospital in Lebanon.

## Methods

### Data sources and study population

The study used de-identified data extracted from the ED discharge database of one of the largest tertiary hospitals in Lebanon. Administrative (patient gender, age, marital status and admission status, categorized payment amounts) and medical (severity index) information was used. Study population comprised 95,879 records representing 60,414 unique patients that visited the ED between July 31, 2012 and July 31, 2014.

### Data analysis

Patient’s bills per record were received from the billing services of the hospital on an excel sheet. The data file also included patients age, gender, file number, billing number, Emergency Severity Index (ESI) score, and disposition (admitted, discharged, or discharged against medical advice (AMA). The ESI was developed by two emergency department physicians [22]. The ESI was conceptualized to integrate patient acuity, and if stable, potential resource needs. ESI levels 1 and 2 indicate high acuity where levels 3, 4 and 5 are for lesser acuity cases. Patient visit bills were reported under six major categories: 1) solutions (including blood bank, medical supplies), 2) pharmacy, 3) laboratory, 4) physicians (medical and surgical), 5) facility (patient and room service), and 6) radiology. The charges, originally reported in Lebanese pounds (LBP), were transformed in US dollar values (1$ = 1500). Miscellaneous billing category was excluded from the analysis as it is frequently used by the billing department for balance purposes to round to the nearest 0.5 dollar amount. A total was calculated for the six categories. It is important to note that analyzed charges only included those for service delivered in the ED (in case of admission, the charges were not part of the analysis). The excel file was then exported to SPSS for further analyses. Characteristics of ED visits were summarized according to calendar year, patient gender, age groups, ESI score, and disposition, using frequencies and percentages. In further analyses, and to focus on average differences of charges among different groups, all independent variables were turned into dummy variables with a specified reference group. This approach allows the analysis to produce estimates of the charge difference between group categories (female vs male for instance). Analysis addressing the association of each of the independent variables (age, gender, ESI score, disposition, and coverage) with the total charges and each of its 6 components was done using simple linear regression (equivalent to the independent *t* test and ANOVA). Multiple regression was conducted with total charges considered as the dependent variable. The unit of analysis was visit to the ED and as such multiple visits during the study period were allowed.

Ethical approval for the study was granted by the Institutional Review Board (IRB) at the American University of Beirut (AUB).

## Results

Females represented approximately half of the sample (48.4 %). Mean age was 34 years with half of the sample below 30 years (27.5 % 0–18 years and 23.7 % 19–29 years) and 17.5 % 60 years of age and above. The average number of visits was 1.6 with a maximum of 64 visits. The majority had an ESI score of 3 (71 %) followed by a score of 4 (22.4 %), and only a minute proportion had a score of 1 (0.3 %). The proportion that was discharged was equal to 82 %; 14.9 % were admitted and 3 % were discharged AMA (Table 1).

**Table 1:** Characteristics of visits of study sample

	Total	
	N	%
Total number of visits	95,879	100.0
Gender		
Female	43,000	48.4
Male	45,821	51.6
Age		
0-18	24,414	27.5
19-29	21,045	23.7
30-60	27,826	31.3
60+	15,553	17.5
Mean (SD)	34.32	(24.1)
ESI		
1	307	0.3
2	3,752	4.2
3	63,119	71.0
4	19,874	22.4
5	1,786	2.0
Disposition		
Admitted	13,194	14.9
Discharged	72,943	82.1
AMA	2,701	3.0

*ESI* Emergency Score Index

*AMA* Against Medical Advise

**Fig. 1:**
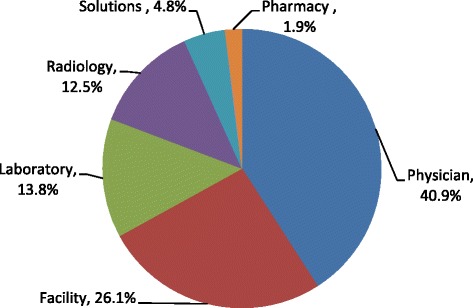
Distribution of ED charges

**Fig. 2:**
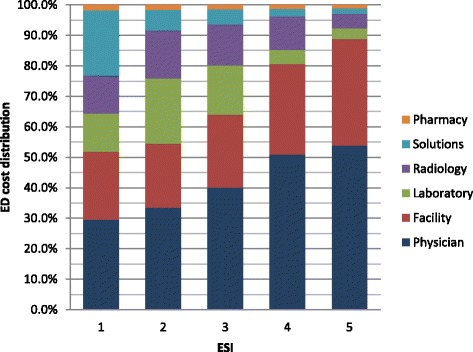
ED charges distribution by ESI scores

**Fig. 3:**
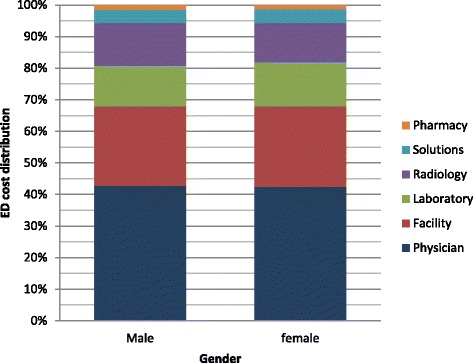
ED charges distribution by gender

**Fig. 4:**
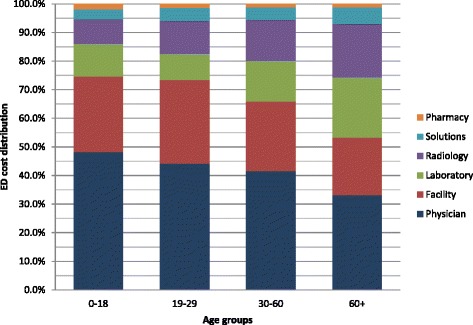
ED charges distribution by age groups

Physician charges represented the highest proportion of total charges (40.9 %), followed by facility that constituted 26.1 % of total charges, then laboratory (13.8 %) radiology (12.5 %), solutions (4.8 %) and pharmacy (1.9 %) (Fig. 1). The distribution of charges by ESI gender and age is presented in Figs. 2, 3 and 4. Charges varied significantly according to ESI score. The contribution of physician charges to the total charges was the highest for ESI 5 patients (54 %), and decreased gradually with descending ESI scores (51, 40, 33.5, and 30 %). Facilities percent contribution displayed a similar pattern (35 % for ESI 5 and down to 22.4 % for ESI 1). The contribution of laboratory charges on the other hand was minimal for ESI 5 (3.3 %) and highest for ESI 2 (21.4 %). A similar pattern was observed for radiology (4.8 % for ESI 5 and 15.9 % for ESI 2). Solution contribution was highest for ESI 1 (21.3 %) and was significantly lower among other ESI scores (6.6 % ESI 2, 4.9 % ESI 3, 2.5 % ESI 4, and 1.8 % ESI 5). The contribution of pharmacy charges, on the other hand, did not vary much with ESI score (1.9 % for ESI 1 to 1.1 % for ESI 5).

Table 2 displays the results of the association of the independent variables with total charges and the six categories for charges. Total charges for males was found to be higher than females by an average of $13 across all categories (all *p*-values < .001), with the exception of laboratory whereby females were charged on average $1.4 more than males (*p*-value = .017). Age was also found to be associated with increasing charges, with older patients receiving higher charges for most of the categories. The total charge among 0–18 years old was lower than the 19–29 years old by $7, but then it increased for age group 30–60 years by an average of $58, and $188 for those above 60 years. The older age groups received higher charges for solutions ($22) laboratory ($63) and radiology ($84). On the other hand, physician charges were highest for 0–18 years old group and lowest for 19–29 years (lower by $17) (*p*-value < .001). The reference group for ESI was a score of five, and consistently the highest charge was for the ESI of 1. The difference in total charges increased from $37 with ESI = 2, to more than $400 for ESI = 1 (*p*-value < .001). Patients with an ESI of 1 were charged $127 more than the reference group on solutions, $13 on pharmacy, 65$ on laboratory, $7 on physicians, $31 on facility and $113 on radiology (*p*-value < .001 for all). Admitted patients and those discharged AMA were charged more than those who were discharged regularly with the exception of physicians’ charges. Admitted and AMA were charged $4 and $7 less, respectively on physician fees than those discharged (*P*-value < .001). Overall, the difference in charges were on average almost $250 for admitted and more than $90 for AMA. Admitted were charged more, on average, $31 on solution, $4 on pharmacy, $79 on laboratory, and $78 on radiology. AMA were charged an additional charge of $10 on solution, $53 on laboratory, and $30 on radiology (*p*-values < .001).

**Table 2:** Association of total charges (in $) and components with independent variables

Variable	Total charges		Solutions		Pharmacy		Laboratory		Physician		Facility		Radiology	
	Mean	SD	Mean	SD	Mean	SD	Mean	SD	Mean	SD	Mean	SD	Mean	SD
Gender														
Female	ref		ref		ref		ref		ref		ref		ref	
Male	13.0	1.9	1.7	.5	2.9	.4	−1.4	.6	3.0	.4	.7	.2	5.5	.9
*P-value*	<0.001		0.001		<0.001		0.017		<0.001		<0.001		<0.001	
Age														
0–18	ref		ref		ref		ref		ref		ref		ref	
19–29	−7.0	2.6	3.7	.7	−5.3	.6	−6.3	.8	−17.3	.6	-.2^@^	.3	20.5	1.3
30–60	58.4	2.4	7.5	.6	−4.8	.5	18.3	.8	−5.8	.5	2.6	.3	39.7	1.2
60+	188.4	2.8	22.4	.7	−2.1	.6	62.9	.9	−5.0	.6	7.8	.3	84.2	1.4
*P-value*	<0.001		<0.001		<0.001		<0.001		<0.001		<0.001		<0.001	
ESI														
1	411.4	16.9	127.2	4.3	12.8	3.5	64.7	5.2	7.4&	3.7	30.9	1.8	113.0	8.4
2	272.2	7.9	38.5	2.1	8.5	1.7	90.4	2.5	5.5	1.8	8.4	.9	102.5	3.9
3	151.8	6.5	17.7	1.7	3.4	1.4	55.8	2.1	8.4	1.5	4.3	.7	57.0	3.3
4	37.0	6.8	2.5*	1.8	1.1*	1.5	4.8*	2.1	10.8	1.5	1.5*	.8	19.1	3.4
5	ref		ref		ref		ref		ref		ref		ref	
*P-value*	<0.001		<0.001		<0.001		<0.001		<0.001		<0.001		<0.001	
Disposition														
Admitted	248.2	2.5	31.4	.6	4.3	.5	79.4	.8	−4.2	.5	-.3^!^	.3	78.5	1.3
AMA	91.2	5.2	10.0	1.4	.13^#^	1.2	52.9	1.7	−7.6	1.2	3.7	.6	30.1	2.6
Discharged	ref		ref		ref		ref		ref		ref		Ref	
*P-value*	<0.001		<0.001		<0.001		<0.001		<0.001		<0.001		<0.001	

* ESI4 vs ESI5 *p*-value = 0.168 for Solutions, and *p*-value = 0.460 for Pharmacy, *p*-value = 0.025 for Laboratory p-value = 0.055 for facility, & ESI1 vs ESI5 p-value = 0.042 for Physician, # AMA vs Discharged *p*-value = 0.917 for Pharmacy @ Age 19–29 vs 0–18 *p*-value =0.474 for facility, ! Admitted vs Discharged *p*-value =0.332 for facility

Physician charges represented the highest proportion of total charges (40.9 %), followed by facility that constituted 26.1 % of total charges, then laboratory (13.8 %) radiology (12.5 %), solutions (4.8 %) and pharmacy (1.9 %) (Fig. 1). The distribution of charges varied significantly according to ESI score (Fig. 2). The contribution of physician charges to the total charges was the highest for ESI 5 patients (54 %), and decreased gradually with descending ESI scores (51, 40, 33.5, and 30 %). Facilities percent contribution displayed a similar pattern (35 % for ESI 5 and down to 22.4 % for ESI 1). The contribution of laboratory charges on the other hand was minimal for ESI 5 (3.3 %) and highest for ESI 2 (21.4 %). A similar pattern was observed for radiology (4.8 % for ESI 5 and 15.9 % for ESI 2). Solution contribution was highest for ESI 1 (21.3 %) and was significantly lower among other ESI scores (6.6 % ESI 2, 4.9 % ESI 3, 2.5 % ESI 4, and 1.8 % ESI 5). The contribution of pharmacy charges, on the other hand, did not vary much with ESI score (1.9 % for ESI 1 to 1.1 % for ESI 5).

Table 3 displays the multivariate regression model of total charges. The model explained 14 % of the variability in the total charges (R2 = 14.1 %). According to the model males were on average charged $14 more than females, regardless of the age, ESI score and disposition (*p*-value < .001). Age was associated with an increased charge, whereby charges were $41 higher for age 30–60 year olds (as compared to 0–18 year olds), and $113 for those above 60 years of age (*p*-value < .001). As the ESI score increased from 1 to 5, the average charge increased by $32 (ESI 4), $103 (ESI 3), $171 (ESI 2 and $306 (ESI 1) (*p*-values < .001 for all groups). AMA groups were charged $48 more than those who were discharged, whereas admitted where found to be charged an extra $186, as compared to the discharged group (*p*-values < .001).

**Table 3:** Multivariable linear regression model of ED total charges

	Beta	SE	95 % CI	*P*-value
Gender:				
Male	14.04	1.74	(11.50 – 16.54)	<0.001
Female (ref)	--	--	--	--
Age groups:				
0–18 (ref)	--	--	--	--
19–29	3.40	2.45	(-1.41 – 8.19)	0.167
30–60	41.40	2.29	(36.90 – 45.89)	<0.001
60+	113.81	2.80	(108.31 – 119.30)	<0.001
ESI				
1	306.07	16.08	(274.56 – 337.59)	<0.001
2	170.97	7.54	(156.19 – 185.76)	<0.001
3	103.25	6.25	(91.00 – 115.50)	<0.001
4	32.50	6.41	(19.94 – 45.08)	<0.001
5 (ref)	--	--	--	--
Disposition				
Admitted	186.53	2.65	(181.34 – 191.73)	<0.001
AMA	48.11	5.14	(38.04 – 58.18)	<0.001
Discharged	--	--	--	--

## Discussion

Countries around the world including developing countries like India and Iran as well as developed countries like the US, Germany, France and Australia are experiencing rising ED visit rates [20]. These trends have major implications on health care costs given the high resource intensity of the ED setting and the associated costs of ED visits that have also seen sharp rises in recent years [16]. In spite of the growing concern about rising ED visit charges and the push for increased cost-containment measures, including calls for increased price transparency and more stewardship in ED resource utilization, few studies have evaluated the components and determinants of total ED charges [19, 20].

This study aimed at examining the distribution of ED charges and factors associated with it. Findings revealed that the professional fee (40.9 %) followed by facility fee (26.1 %) accounted for the majority of the ED charges. While Williams et al looked at ED costs rather than charges, their study also found the physicians and facility fee were the major components of ED costs, though the facility fee in their study was a greater contributor at 40.2 % compared to the physician contribution of 30.5 %. Ancillary services in our study accounted for the next major contribution to the ED total charges with Laboratory at 13.8 % and Radiology at 12.5 %. This, too, was the next biggest category in the William’s study though radiology services were slightly larger contributors than laboratory (11.6 % compared to 10.0 %) [21]. While greater than 80 % of the visit charges went to physician and facility fee for low acuity cases (ESI 4 and 5), these contributed to only 52 and 54 % of the high acuity presentations (ESI 1 and 2 respectively) where ancillary services and solutions’ contribution to the total charges increases. This is similar to findings in prior studies and suggests that looking at reduced resource utilization as a cost-containment measure for lower acuity patients may not be effective. Rather, the focus should be on reducing facility and professional fee charges for these patients through triaging to less resource intensive areas within the ED or acute care facility where facility and professional fee charges can be differentiated [21].

Few studies have looked at predictors of ED resource utilization as a reflection of ED resource utilization. While Henneman et al. found no association between resource utilization by gender, except in women of child-bearing age who had higher resource utilization rates, our study found a relatively higher charges for males ($14) compared to females after adjusting for all other variables [23, 24]. Similar to other studies that have found an association between age and resource utilization [23], age was a predictor of higher charges in our study, with total charges of patients greater than 60 years of age being around $113 higher than ages 0–18 after controlling for all other variables. With ED geriatric visits rising globally [25, 26], this has tremendous implications on total health care costs and planning for acute care.

Emergency Severity Index (ESI) is a triaging system that assesses initially for high acuity, then triages patients based on expected resource utilization. Studies have found that ESI not only has very good inter-rater reliability but also accurately predicts ED resource consumption [27, 28]. This study revealed two interesting findings: 1) higher acuity patients had a greater proportion of the ED charges attributed to ancillary services including radiology and laboratory and 2) the lower ESI (higher acuity) the higher the total charges even after controlling for other variables, with ESI 1 charges being $307 higher than ESI 5. Williams (1996) had also showed that the distribution of ED charges is significantly associated with the level of patient urgency. For non-urgent visits, hospital facility and physicians’ costs constituted the bulk of charges, while for urgent medical conditions ancillary services, including laboratory and radiology, contributed more to the total charges [29]. The study also showed that patients who required admission also had significantly higher charges reflecting the increased complexity and resource requirement of admitted patients. Such findings can form the basis for promoting the use of ESI as a proxy estimate of price/charges. This is especially true for many countries of the World where patients, especially out-of-pocket ones, demand to have an estimate of the services delivered/to be delivered to them during their ED visit. This is in line with the global push for price transparency as a means of cost-containment and the challenge of diagnosis based price estimates in the ED setting where patients initially present with undifferentiated symptoms [11, 16].

Though this study was done at an institution that is staffed by American Board Emergency Medicine physicians and uses internationally recognized triaging system, generalizability of our findings is limited by this being a single site analysis that reflects institutional pricing of the different component, and the categorization, of the ED charges as well as existing physician practices. In addition, the timeframe of the study and the limited available variables constrained the ability to delve more into associations of other characteristics with charges. Furthermore, this study is limited to charge analysis and does not evaluate cost to patient and institution.

## Conclusion

As ED utilization and costs increase globally, understanding the components and determinants of ED charges is essential to developing cost-containment interventions. The study demonstrates that multiple variables that can be identified at presentation are tied to higher ED charges including gender, age and ESI. Institutional modeling of historical charging patterns with these variables can be used to offer price estimates to ED patients who request this information and ultimately may help create market competition to drive down costs.
